# O18 The Rollercoaster Reality of Mixed Connective Tissue disease

**DOI:** 10.1093/rap/rkaa054.006

**Published:** 2020-11-03

**Authors:** Maysoon Elfadil, Madeleine Mackay, Daniel Hawley, Lisa Dunkley, Rachel Tattersall, Anne-Marie McMahon

**Affiliations:** r1 Paediatric Rheumatology: Sheffield Children's Hospital NHSFT, Sheffield, United Kingdom; r2 Adolescent and Adult Rheumatology: Sheffield Teaching Hospitals NHSFT, Sheffield, United Kingdom

## Abstract

**Case report - Introduction:**

Childhood-onset mixed connective tissue disease (MCTD) is rare and heterogeneous presenting both diagnostic and treatment challenges. Symptoms often present sequentially over an extended period of time, requiring a high index of suspicion and careful clinical evaluation to diagnose. We present the cases of two young women which demonstrate the evolution of this disease and the diagnostic and management challenges. Summarising these two cases from childhood, through adolescence and into adult life will highlight the importance of integrated clinical pathways and effective transition in managing their disease.

**Case report - Case description:**

Case 1: Twenty-year-old female presented in 2016, age 15 years with polyarthritis, Raynaud’s phenomenon, fatigue, and headaches – rheumatoid-factor (RF) positive, anti-RNP antibody positive, Smith antibody positive at diagnosis. Initially treated with methotrexate, prednisolone, and hydroxychloroquine. Due to methotrexate-associated nausea, she switched to sulfasalazine but remained steroid dependent requiring several doses of methyl prednisolone. Treatment was escalated, and transfer delayed, and in 2017 (age 16) she had rituximab with good efficacy. She transferred into adult rheumatology with careful monitoring for symptom evolution. Her arthritis flared in November 2019, associated with fatigue and malaise, and she developed immune thrombocytopenia (ITP) in April 2020. The combination of arthritis, ITP, malaise, and activation of complement (without other features of lupus) necessitated a further pulse of rituximab with resolution of symptoms and normalisation of complement.

Case 2: Twenty-two-year-old female presented in 2006, age 9 with fatigue, polyarthritis, leucopoenia, and positive ANA. Managed as JIA with prednisolone, methotrexate and etanercept then switched to infliximab as features of juvenile dermatomyositis developed. Arthritis and muscle weakness worsened with Raynaud’s phenomenon developing and new sclerodactyly. Serology evolved with positive ANA, anti-RNP antibodies and positive RF leading to diagnosis of MCTD. Treatment included azathioprine and mycophenolate mofetil (MMF). Disease escalated and cyclophosphamide/rituximab administered. Remission was reached in 2011, age 13, and maintained with rituximab, methotrexate, and hydroxychloroquine. Transition into adult services was carefully planned and transfer occurred in a period of remission age 17. In adult services she is stable on azathioprine and hydroxychloroquine and sclerodactyly has completely regressed. Treatment complications are evident -panhypogammaglobulinaemia secondary to rituximab treatment and steroid induced osteoporosis/fracture.

**Case report - Discussion:**

MCTD was first suggested as a distinct entity in 1972 by Sharp and colleagues. There is a lack of consensus over the four published diagnostic criteria (Kasukawa’s, Alarcon-Segovia, Kahn and Sharp), none of which have been validated for use in children. Heterogeneity of clinical features and the rarity of childhood-onset MCTD makes diagnosis a challenge. Raynaud’s phenomenon and polyarthritis are the most common presenting features. It was only with time that the clinical picture of MCTD emerged. There is limited evidence available to guide treatment but emerging evidence of benefit from rituximab. Management aims to minimise active inflammation and treatment toxicity balancing the evolving clinical picture with side effects from treatments used, as exemplified in these two cases. Given potential evolution to SLE anti-TNF drugs are usually avoided but in case 2 when this appeared phenotypically to be JIA/JDM both etanercept and infliximab were used.

In Sheffield transfer usually occurs around the end of school year 11 but for case 1 this was the time of maximum clinical instability. Transition was delayed enabling treatment with rituximab in a familiar environment and transfer only occurred when disease was stable. In case 2 with such intensive treatment in paediatric rheumatology transition planning carefully emphasised the clinical features and medicines used which has been of great benefit in adult services to maintain disease control and quiescence and avoid risks of over treatment. An integrated transition pathway from paediatric to adult services has ensured a safety net to the evolving nature of these inflammatory diseases and allowed a joint approach to the diagnosis and management.

**Case report - Key learning points: Evolution of disease:**

Paediatric-onset MCTD is rare. As paediatric and adolescent rheumatologists we will be seeing patients early in the disease course. We need an awareness of the potential for our inflammatory patients’ symptoms to evolve and to re-assess the diagnosis when this happens.

**Respect anti-RNP antibodies:**

anti-RNP antibodies, either at disease outset (case 1) or occurring later in disease course (case 2) help distinguish evolving MCTD, presenting with polyarthritis with or without Raynaud’s phenomenon, from ‘straightforward’ polyarticular JIA. Children and young people who have anti-RNP antibodies need to be watched carefully for evolution of MCTD symptoms.

**Integrated Transition:**

As MCTD may evolve over several years, a close partnership between paediatric and adult rheumatology (whether part of an integrated adolescent transition service or not) helps deliver developmentally appropriate health care.

**Expect complications:**

Paediatric onset MCTD tends to have a milder disease course than in adults, although complete remission is rare. The most common causes of death are linked to pulmonary hypertension and interstitial lung disease. However, these complications are less seen in children. Clinical vigilance and proactive screening in watching for development of the disease and trying to anticipate potential problems are important. This requires multi-disciplinary team working. This is just as important when managing treatment effects such as panhypogammaglobulinaemia and liaison with immunology team or steroid side effects and liaison with endocrinology team.

**Individualised Approach:**

There is no one specific treatment for MCTD. The heterogeneity of the condition dictates an individualised approach using best available evidence. It is particularly important to review treatment strategies as new symptoms emerge, or side-effects emerge from treatments being used.

